# Bridging the gap: a Canadian perspective on translational kidney research

**DOI:** 10.1186/s40697-014-0018-5

**Published:** 2014-08-29

**Authors:** Amber O Molnar, Darren A Yuen, Navdeep Tangri, Victor L Jensen

**Affiliations:** Kidney Research Centre - Ottawa Hospital Research Institute, University of Ottawa, Ottawa, Canada; Division of Nephrology, The Ottawa Hospital, Ottawa, Canada; Division of Nephrology, St. Michael’s Hospital, University of Toronto, Toronto, ON Canada; Keenan Research Centre of Biomedical Science of St. Michael’s Hospital, Toronto, ON Canada; Section of Nephrology, Seven Oaks General Hospital, University of Manitoba, Winnipeg, MB Canada; Molecular Biology and Biochemistry, Simon Fraser University, Burnaby, BC Canada

**Keywords:** Translational research, Kidney, Renal, Funding, Therapy, Cystic, CKD, KFRE, AKI, KRESCENT

## Abstract

**Purpose of review:**

Chronic kidney disease affects approximately 3 million Canadians. Ongoing investment in high quality kidney research is needed to improve the care of patients with kidney disease. The barriers to translating such research are discussed in this review.

**Sources of information:**

Personal knowledge, research funding body websites, and published reports.

**Findings:**

In this review, we discuss the meaning of the term translational research and present some of the programs aimed at ensuring efficient translation of scientific discoveries with a discussion of the barriers to translation. We highlight some successes and barriers to kidney research translation using recent examples of research in Canadian nephrology. We present the following examples of kidney research: (1) research aimed at identifying the causative genes for inherited kidney diseases; (2) recent discoveries in cell-based therapies for kidney disease; (3) an examination of the impact of acute kidney injury in renal transplant patients; and (4) the development of a kidney failure risk equation to improve prognosis accuracy.

**Limitations:**

This review focuses on research conducted by the authors.

**Implications:**

The process of research translation is prolonged and challenging and therefore requires resources, patience, and careful planning. With increased awareness and understanding of the barriers to research translation, researchers and funding bodies can work together to increase the rate at which important research findings reach clinical practice and improve the care of patients with kidney disease.

## Abrégé

**Objectif de l’étude:**

La néphropathie chronique touche environ 3 millions de Canadiens. Un investissement soutenu dans la recherche de haute qualité en néphrologie est nécessaire à l’amélioration des soins aux patients. Dans cette étude, nous abordons les obstacles à l’application de ces recherches.

**Sources d’information:**

Les connaissances personnelles, les sites Web d’organismes de financement de la recherche, et les rapports publiés.

**Résultats:**

Dans cette étude, nous traitons de la signification du terme « recherche translationnelle » et présentons certains des programmes visant à assurer une circulation efficace des découvertes scientifiques, en abordant les obstacles à la circulation et à l’application. Nous présentons des réussites de circulation de la recherche en néphrologie, de même que certains obstacles, en recourant à des exemples récents de recherche canadienne en néphrologie. Nous citons les exemples suivants de recherche en néphrologie: (1) la recherche visant à déterminer les gènes responsables des néphropathies héréditaires; (2) les découvertes récentes en matière de thérapies cellulaires pour les néphropathies; (3) l’examen des conséquences d’une insuffisance rénale aiguë chez les patients ayant subi une transplantation rénale; et (4) l’élaboration d’une équation concernant le risque d’insuffisance rénale afin d’améliorer la précision du pronostic.

**Limites de l’étude:**

L’étude se concentre sur les recherches effectuées par les auteurs.

**Conséquences:**

Le processus de circulation et d’application de la recherche est long et ardu, si bien qu’il requiert des ressources, de la patience et une planification étroite. Grâce à une compréhension et une sensibilisation accrues des obstacles à la circulation de la recherche, les chercheurs et les organismes de financement peuvent travailler ensemble afin d’accroître le rythme avec lequel les importantes conclusions de recherches atteignent la pratique clinique et améliorent les soins aux patients atteints de néphropathies.

## What was known before

There is a rising awareness of the importance of research translation in the research community. Funding bodies are recognizing that increased resources are needed to improve the success rate of translating research findings into clinical practice.

## What this adds

Through specific examples of Canadian nephrology research, the process of research translation is presented, with a particular emphasis on the barriers to translation.

## Lay summary

What is translational research? Often this term is synonymous with “bench-to-bedside”, meaning to find a way to transform laboratory research into new medicines or treatments for patients. Going one step further, translational research also involves making sure that health care providers are consistently providing recommended treatments to patients. This review discusses the meaning of translational research within the context of kidney research. We also highlight the barriers that prevent the movement of research findings from the laboratory into clinical practice. Funding bodies and agencies have given more resources to translational research and have established specific programs to this end. This will hopefully help overcome the barriers of research translation. Here we discuss, Canadian research, funded by Canadian taxpayers and donors and performed in the context of a Canadian national training program, with an emphasis on the translational aspects of our work. Included are looks at a basic science approach to identifying inherited diseases with kidney involvement, cell-based therapies for kidney disease, an examination of the factors involved in deciding a treatment plan following acute kidney injury, and consideration of risk-based care for patients with chronic kidney disease. Where do we go next? How do we ensure the findings of current kidney research are extended into clinical practice? The road ahead looks promising as funding agencies stress a balanced approach for both basic science and clinical research, and a focus on programs directed at translating research findings.

## Introduction

Translational research has become a popular term in the research community, and may have different meanings depending on the specific situation. When using the term translational research, many are referring to the concept of “bench-to-bedside” where knowledge from basic science is used to produce new drugs and treatment options for patients. For others, translational research refers to translating research into clinical practice, also known as knowledge translation, meaning: “the new treatments and research knowledge actually reach the patients or populations for whom they are intended and are implemented correctly [1]”.

The Institute of Medicine’s Clinical Research Roundtable originally described the model of translational research as a two-phase process progressing in the first phase from basic science to clinical research. They named this first phase T1. The second phase, also known as T2, was defined as the progression from clinical science to everyday clinical practice and health decision-making [1,2]. In 2011, Drolet *et al*. further expanded on this model of translational research and developed what they called the Biomedical Research Translation Continuum, with three “translational chasms”. The first translational chasm (known as TI) represents the transition from basic science research to a proposed human or clinical application. Translational chasm 2 (T2) represents the transition from the proposed human application to a proven clinical application, i.e. evaluation of safety and efficacy using clinical trials. Finally, translational chasm 3 (T3) is the adoption into clinical practice or essentially knowledge translation [3]. The recent recognition of the importance of T3 has led to a growing area of practice-based research. This research can be used to determine if research findings are being adopted by clinicians and having an impact on public health. If practice-based research demonstrates that clinicians are not changing practice, then there has been a block at T3, and one must determine why the block is occurring.

Moving laboratory discoveries into the clinical realm followed by the ultimate goal of a change in clinical practice or public policy can prove to be extremely challenging with many barriers along the way. A study that reviewed articles published in leading basic science journals found that approximately 25% of articles that reported promising findings resulted in the publication of a randomized controlled trial, and less than 10% of the promising findings were introduced into clinical practice over a period of 20 years [4]. It can frequently take several years to bring discoveries made in the laboratory to the general public. In fact, studies that examined the average time from publication of a research finding to adoption into clinical practice have found that the process takes on average 17 years, a figure that has not changed much over the past century [5]. Also, a study conducted in the United States that assessed health care delivery and utilization by telephone survey and chart review found that only 55% of patients receive recommended care [6]. Taken together, these statistics illustrate that promising research findings rarely make it into clinical practice, and when they do, there is a significant lag time between discovery and implementation.

So why does the translation of research prove to be so difficult? Sung and colleagues refer to the barriers of translation as “translational blocks”, which can include financial constraints, regulatory agency burden, fragmented infrastructure, or a lack of qualified investigators [2]. If not anticipated and without the appropriate resources in place, these barriers will all result in a lack of research translation.

Despite the concept of translational research being introduced over 30 years ago [7], the subject has only become a major focus over the past decade. This is illustrated by a sharp rise in publications on the subject, with the number of publications increasing from only 5 in 1994 to 110 in 2007 [8]. Along with a large number of publications in the literature, research funding bodies and training programs have recognized the need in recent years to make translational research a priority.

The Canadian Institutes of Health Research (CIHR) has made translating knowledge from the research setting to the public a key component of its mandate, with funding and educational programs specifically dedicated to knowledge translation [9]. The CIHR defines knowledge translation as: “a dynamic and iterative process that includes synthesis, dissemination, exchange and ethically sound application of knowledge to improve the health of Canadians, provide more effective health services and products and strengthen the health care system”. A large driver of the emphasis on knowledge translation by the CIHR is the increased focus on research governance and accountability. The CIHR considers it a priority that taxpayers’ dollars invested into health research result in some sort of impact on patients [10].

The National Institutes of Health (NIH) established a National Center for Advancing Translational Sciences (NCATS) in 2011. Within the NCATS is the Clinical and Translational Science Award (CTSA) program, which supports a national consortium of more than 60 medical research institutions. Funding of NCATS has increased on an annual basis, and the requested budget for 2014 is over $665,000,000 [11]. The European Commission has also followed suit, allocating most of its 6 billion Euro health research budget for 2007–2013 to pan-European translational research projects. There are also a growing number of programs across Europe that offer training in translational research [12].

A kidney specific, national research program within Canada known as the Kidney Research Scientist Core Education and National Training (KRESCENT) program has also made knowledge translation a top priority [13]. The KRESCENT program was founded in 2004 and is headed by Dr. Kevin Burns, a nephrologist and basic scientist with the University of Ottawa and Dr. Adeera Levin, a nephrologist and clinical research scientist with the University of British Columbia. KRESCENT is a multi-partner collaboration founded by the Canadian Society of Nephrology, CIHR and the Kidney Foundation of Canada. The program emphasizes translational research by funding research fellowships to individuals from various disciplines. Members of the program range from dietitians to PhD scientists to nephrologists. The program meets on a bi-annual basis and members share their diverse research. The concept of “bench-to- bedside” is a focal point of the discussion [14]. Fellows within the KRESCENT program complete their research training equipped with the skills for research translation. This skill set is provided formally by guest speakers at the bi-annual meetings, assignments and grant writing seminars; and informally by the program heads and collaboration with other fellows in the program.

There is clearly rising awareness and funding for research translation. We present in this review four examples of recently published and ongoing Canadian translational research pertinent to nephrology. The research presented spans the continuum of basic science, clinical application, and public health policy, illustrating the breadth of work being done by the Canadian renal community. More importantly, these examples demonstrate that translation of research is not just a unidirectional process, but rather provides opportunities to move bidirectionally between the bench and the bedside to ask and answer important questions relevant to the kidney. The examples presented also highlight some of the key barriers to successful research translation.

## Genetic disorders with renal involvement and cilia

Investigation of fundamental biological processes has provided critical insights into our understanding of how organs behave in health and disease. Specifically, interrogation of the phenotypic manifestations of gene mutations has informed our understanding of multiple diseases. In this regard, the kidney is no exception, as many genetic disorders have some level of renal involvement [15–17]. Whether these disorders solely affect the kidneys or multiple tissues within the body with accompanying renal dysfunction, often there are high rates of renal morbidity. Among these genetic disorders are the cystic kidney diseases polycystic kidney disease (PKD) and nephronophthisis (NPHP) [17,18]. There are monogenic diseases that cause solely PKD or NPHP, but they can also occur as part of a more broad disorder such as Bardet-Biedl, Joubert, Senior–Løken, and Orofaciodigital syndromes where end stage renal disease (ESRD) is quite common [16–18]. The above listed diseases are among ciliopathies, where the molecular pathology is rooted in the cellular organelle, the cilium. Cilia are microtubule-based organelles that protrude from the surface of the cell with gated cytoplasm and membrane [17–19]. Primary (or non-motile) cilia are concentrated in several signaling pathways and serve as major sensors for the extracellular environment. In kidneys, primary cilia are present on tubule epithelial cells and are mechanosensory as well as chemosensory, in that they sense urine flow and the presence of signaling molecules [20]. Dysfunction in cilia, either from loss or alteration of a cilia-related gene, can lead to the above diseases, often resulting in over-proliferation of renal tubule cells and ultimately, cystogenesis [18,20].

For many ciliopathies, researchers involved in basic scientific studies will have first characterized the causative gene. One example, is with IFTA-1 (intraflagellar transport subcomplex A – 1), studied in the model organism *Caenorhabditis elegans* [21]. In *C. elegans*, the IFTA-1 protein was shown to be a component of the IFT complex, a strong indication that mutation of the human ortholog WDR35 would likely result in typical ciliopathy phenotypes. Subsequently, two studies showed that mutations in IFTA-1/WDR35 (WD repeat protein 35) were causative in Sensenbrenner and short-rib polydactyly syndromes, both of which include cystic kidneys [18,22,23]. Previous characterization of a gene in model organisms can assist in narrowing in on causative mutations, as it did for IFTA-1/WDR35.

Another type of connection between basic and clinical science can come in the form of a comprehensive study of a novel gene. The discovery that TMEM237 (transmembrane protein 237 or JBTS14, Joubert syndrome 14) harbored causative mutations in ciliopathy patients provides such an example [24]. Common characteristics of Joubert syndrome include the molar tooth sign, hypotonia, ataxia, hyperpnea, and cystic kidneys [24]. This extensive study not only included data from the sequencing of Canadian and Austrian patients, there were also functional studies of the TMEM237 performed in *C. elegans*, *Danio rerio* and cultured mammalian cells. Together, these data provided a broad look into the function of this gene and the consequences of its loss on human health. One immediate impact from this discovery for patients and their families is genetic testing for patients to assist in diagnosis and for family members to identify disease mutation carriers. Two of the three equally contributing laboratories are from Canada.

Though the discovery of candidate and novel ciliopathy genes affects a relatively small number of patients directly, there is the potential for a broader impact. Immediate impacts include: enhanced genetic counseling for families of a diagnosed proband, faster or more accurate diagnosis through sequencing, and – though controversial – the potential for pre-implantation genetic screening [18]. Though genetic testing can identify a particular disease (or identify carriers of disease-causing mutations), the possibility of negative consequences exists, such as inability to obtain insurance coverage or potential negative psychological impact [25]. For example, six laboratories currently offer genetic testing for TMEM237/JBTS14, which is discussed above [26]. Another benefit derived from the deeper comprehension of the molecular pathology of each disease is the enriched understanding of human biological processes that may lead to targeted therapies.

Though loss or mutation of ciliary components leads to many disorders, cilia may also play a role in acute kidney injury. It has been shown in transplant patients as well as in mice that upon kidney injury, cilia become significantly shorter [27–31]. After the injury, during the repair phase, cilia were longer than in controls. The mechanism for this length control remains elusive and no role has been found for cilia or cilia-related signaling in kidney injury or subsequent recovery. Despite this, it is entirely possible that cilia do play a role in kidney injury and recovery because they function as the environmental sensor of the cell, sensing the surrounding conditions [30]. Changes in cilia length have been observed for many of the monogenic ciliopathies (and/or the corresponding model organisms) [18,20,32]. Because ciliopathy genes and kidney injury both result in alteration of cilia length, it is possible that common variants in these genes may alter ability and/or rate of recovery from kidney injury as well as potentially aiding in prediction of a patient’s prognosis. Likewise, if the role of cilia in recovery from kidney injury can be elucidated, there is the potential that it will aid in the discovery of targeted therapeutics. Therefore, research into monogenic kidney disorders may not only impact patients with these diseases, but also has the potential to enhance treatment of other disorders (Table 1).

**Table 1:** **Future opportunities for research translation**

**Topic of research**	**Opportunities for research translation**	**Translational chasm**
Discovery and characterization of novel ciliopathy genes	• Genetic counseling for families.	Moving from basic science to a proposed human/clinical application (T1)
	• Accurate diagnosis of a specific condition using genetic sequencing.	
	• Although controversial, there is the potential for pre-implantation genetic screening.	
	• Potential for the development of targeted therapies (genetic disorders and acute kidney injury).	
Cell based therapies for kidney disease	• Potential for a novel therapy for the treatment of progressive CKD. Design of a randomized controlled trial to test the infusion of EOC derived factors vs. standard care for the treatment of CKD.	Moving from basic science to a proposed human application (T1) and now towards a proven clinical application (T2)
Acute kidney injury in the renal transplant population	• Development of a predictive model that would predict the risk of graft loss or death following an episode of AKI.	Moving a clinical research finding into clinical practice (T3)
	• Potential to use the developed predictive model to identify high risk patients for the study of mechanisms of graft loss. Mechanistic studies could lead to targeted therapies for testing in randomized controlled trials.	Moving a clinical research finding back to basic science and towards a proposed human application (T1)
Risk based care for chronic kidney disease	• Dialysis access planning pathways that incorporate the KFRE.	Moving a clinical research finding into clinical practice and performing practice based research (T3)
	• Cost utility analyses of referral and treatment pathways that incorporate the KFRE.	
	• Evaluation of the KFRE against novel biomarkers for CKD progression.	

While elucidating the role of cilia in kidney disease is an exciting and fascinating topic, this research is still far from the end goal of changing clinical practice. This research is still primarily at the basic science level and will need to overcome the barrier of transitioning to a proposed human or clinical application (T1). Presently, the primary human or clinical application of this research is genetic screening or counseling, which has its own barriers to widespread adoption into clinical practice due to concerns about cost and consequences for the patient, such as the ability to obtain insurance coverage.

## Cell-based therapies for kidney disease

As discussed above, the study of fundamental biologic processes can provide important insights that ultimately inform prognostic and/or therapeutic applications of these discoveries. In most cases, as knowledge is translated towards models that more closely reflect human health and disease, further concept refinement is often required. An example of this refinement process is illustrated by the challenges that investigators have faced in translating exciting discoveries made more than 20 years ago suggesting that cell-based therapies have the potential to attenuate or even repair chronic organ injury. In the 1980’s and 1990’s, a number of groups described the presence of bone marrow-derived cell populations with the ability to regenerate and/or repair injured organs [33–35]. Recognizing the therapeutic potential of these fundamental discoveries, investigators began testing whether such cells could be grown in the lab and used as either a preventative or regenerative treatment for chronic injury of the kidney and other organs.

As these cells were originally thought to work by replacing lost or damaged cells in injured tissues [33,36–40], initial studies focused on complex and often highly invasive infusion strategies to maximize delivery of cells to the injured organ, such as direct injections into feeding arteries [37,41,42]. While such studies demonstrated often dramatic protective effects of cell infusion, enthusiasm for such strategies as a clinical treatment was tempered by the invasive nature of the therapy [43]. Around the same time, concerns were also being raised regarding the safety of infusing bone marrow-derived cells, with some studies suggesting the potential for exacerbation of tissue injury [44,45], and others even reporting uncontrolled growth of such cells following infusion [46]. Finally, other reports suggested that diseases such as diabetes and chronic kidney disease (CKD) could impair the tissue protective activity of these cells [47–54], rendering an autologous cell treatment strategy for CKD less attractive.

In an effort to overcome these barriers, a number of investigators made a key discovery. In most cases, infused bone marrow-derived cells are not retained in significant numbers in the injured organ despite exerting potent protective effects [55–57]. For example, Dr. Yuen and colleagues demonstrated that treatment with early outgrowth cells (EOCs), a type of bone marrow-derived cell that can be grown in large numbers in the lab, was able to dramatically reduce progression of experimental diabetic and non-diabetic CKD. Intriguingly, Dr. Yuen and his colleagues demonstrated that following infusion, EOCs homed to organs of the reticuloendothelial system, such as the liver, spleen, and bone marrow [55–57]. This finding suggested that infused EOCs might lodge in these organs, working by secreting soluble factors that act in an endocrine fashion to protect and repair the injured kidney. Subsequent studies revealed that EOCs and other bone marrow-derived cells do, in fact, release soluble factors that can mediate a wide variety of beneficial effects *in vitro* [55,57–60]. The activity of these soluble factors could be maintained even with extensive dilution, suggesting that these factors could, in fact, work via an endocrine mode of action [61].

The potency of these factors also suggested that they might be used directly as a therapy for kidney disease without the need for, and associated risks of, cell infusion. In this regard, a recent proof-of-principle study confirmed that infusion of a cell-free preparation of EOC-derived factors significantly reduced renal injury and dysfunction in rats with experimental CKD, mimicking the effects of cell injection [61]. Follow-up studies have further demonstrated that EOC derived factors have additive renoprotective effects on top of renin-angiotensin system blockade [62].

Taken together, this work points to the exciting potential of cell-based treatment for kidney disease and provides an example of how the iterative process of translational research can rapidly refine and advance a novel treatment from the bench to the bedside. Moving forward, Dr. Yuen is part of a Canadian group of investigators that is currently planning a clinical trial comparing EOC-derived factors against standard of care therapy in high risk, proteinuric chronic kidney disease patients who continue to progress on renin-angiotensin system blockers. While such a trial would provide important proof-of-concept data supporting a cell-based treatment strategy for CKD, the long term goal is to identify the specific factor(s) responsible for the potent renoprotective activity of EOC therapy. Importantly, the identification of these factors would allow potential enhancement of renoprotection and would alleviate safety concerns regarding the administration of unknown proteins that do not contribute to renoprotection. These promising pre-clinical results have formed the basis for a proposed clinical trial of EOC-derived factors in patients with CKD (Table 1).

Now that this basic science research has overcome the barrier of finding a proposed human or clinical application (T1), the next step is to understand if this translates into clinical safety and effectiveness with phase 1, 2 and 3 clinical trials (T2).

## Acute kidney injury

There are several studies in the general population demonstrating that acute kidney injury (AKI) requiring dialysis is associated with an increased risk of death and chronic kidney disease [63–66]. There is also increasing evidence that less severe AKI is associated with an increased risk of poor long-term outcomes [67–71]. However, the impact of AKI on outcomes in the renal transplant population is less well characterized. We are aware of two recently published studies that have examined this issue. Both studies showed that, similar to the general population, renal transplant patients who experience AKI have a heightened risk of poor long-term outcomes [72,73]. With an association between AKI and poor outcomes among renal transplant patients now being demonstrated, how can this discovery be translated to patients and clinical practice?

Further characterization of the association would first be needed. It remains unclear if all transplant patients who develop AKI experience worse outcomes. Potentially certain patient characteristics or characteristics of the AKI event modify the association, and if so, which characteristics determine a worse prognosis. We propose to design a study that would examine the association of key patient and AKI event characteristics with graft loss and death. The characteristics examined would be easily ascertainable from large existing health care databases. The knowledge gained from this research could be used to develop a predictive model that could serve as a helpful tool for clinicians to identify transplant patients at high risk for poor long-term outcomes following an episode of AKI. Identifying high-risk renal transplant patients at hospital discharge could lead to a practice change of selected patients being followed more closely. It is our hope that identifying high risk patients would lead to improved patient outcomes.

However, there are several potential barriers to this research. The first barrier is to successfully design and conduct the study. The creation of a robust predictive model requires a large sample size, and patients with a kidney transplant are relatively small in number. The next limitation is that of the database itself. Due to the follow up time required for the study, and the resources involved with creating a prospective database across transplant centres, we will utilize already existing health care databases. Given that this will be a retrospective study, only variables readily available in the database can be included. The variables included in the model are also very important when considering the clinical application and uptake into clinical practice. Clinicians will want a predictive model that is easy to use and incorporates variables that are readily available. Successful implementation of the model into clinical practice will also require a knowledge translation plan.

It is our hope that simply identifying high-risk patients with the model and following them more closely will improve outcomes; however, this may not be the case. Following our development of the predictive model, an algorithm or management strategy for high-risk patients will need to be implemented and applied to clinical practice. Further research will then be needed to test whether a change in management of high-risk patients results in a change in clinical endpoints.

The information gained from this study could potentially be used to move from the bedside back to the bench. The ability to identify high-risk transplant patients could facilitate the conduct of basic science studies examining the mechanism by which AKI leads to death and graft loss. Mechanistic studies could potentially lead to new therapies for testing in randomized controlled trials (Table 1).

## Risk based care for chronic kidney disease

An estimated 3 million Canadians suffer from CKD and are at risk for cardiovascular events and progression to kidney failure [74]. While all patients with CKD are at increased relative risk of kidney failure and all-cause mortality when compared to individuals without CKD, the risk for these adverse outcomes can vary greatly, even for individuals with the same level of estimated glomerular filtration rate (eGFR)/kidney function [75–77]. For interventions such as vascular access insertion, applying the same treatment approaches for patients at high risk for kidney failure to those at low risk can result in unnecessary cost and potential harm [78]. Even routine interventions such as renin-angiotensin aldosterone system (RAAS) inhibitors may have diminished benefits for preventing CKD progression in patients without albuminuria and higher risks of acute kidney injury and hyperkalemia in the same individuals when compared to other antihypertensive agents [79,80]. In order to accurately estimate the risk of kidney failure requiring dialysis or transplant in an individual patient, we developed the kidney failure risk equation (KFRE) in 2011 [77].

The KFRE was developed and validated in 8,931 patients with CKD Stages 3–5 referred for nephrology care in the provinces of Ontario and British Columbia. We developed several equations, but our preferred KFREs (4 variable and 8 variable), were entirely laboratory based, and predicted the outcome of kidney failure in a 2 to 5 year horizon with a high degree of accuracy. These equations will be published with accompanying risk calculators for use on personal computers, tablets and on every smartphone platform. Links to download the individual applications, and the Microsoft Excel version of the risk calculator were provided as part of the original publication, and greatly accelerated the knowledge to action cycle for the KFRE [77,81].

Concordantly, the KFRE has been validated externally by several independent investigators and is used routinely for the triage of nephrology consultations and dialysis access planning in advanced CKD clinics in multiple jurisdictions [82–84] (and unpublished observations). In particular, the province of Manitoba has adopted a centralized triage process for incoming nephrology consultations, where all consults are risk stratified by the KFRE, and those with a < 3% risk of kidney failure in the next 5 years are referred back to primary care, with instructions to re-refer if kidney function declines. The adoption of this referral strategy has resulted in a significant reduction in wait times for nephrology care (10 months to 2 months), and has thereby increased patient and provider satisfaction for high risk referrals (unpublished observations). Similar strategies in other jurisdictions have resulted in better vascular access planning and more informed treatment modality discussions in older patients with CKD [84]. Together, these global and local knowledge translation strategies have greatly increased the impact of the KFRE, and represent an example of translational research from the epidemiology bench to the health policy bedside (personal communication, Daniel Schwarz).

Future directions for knowledge translation of the KFRE include implementation in dialysis access planning pathways, cost utility analyses of referral and treatment pathways that incorporate risk, and evaluation of the KFRE against novel biomarkers for CKD progression (Table 1).

The largest barrier for this research to overcome is the widespread uptake into clinical practice (T3). The successful uptake by clinicians will require funding and strategic planning for knowledge translation. Successful widespread use of the KFRE will likely require a knowledge translation action plan followed by repeated follow up on the results of the action plan with modifications as needed. These studies will likely include decision analyses based on real data, as well as cluster randomized trials or time series analyses measuring the effects of knowledge translation.

## Conclusions

This review highlights four areas of research in Canadian nephrology conducted by trainees in the KRESCENT program. The translational nature of the research highlights the success of the KRESCENT program. The diverse areas of expertise of the researchers highlight the unique opportunity for collaboration among basic and clinical scientists afforded by the KRESCENT program. In this review, important discoveries in the area of nephrology and successful examples of research translation are presented, such as the development of the KFRE influencing clinical practice and thus improving the care of renal patients locally in Manitoba. On the other hand, many barriers to research translation are illustrated in the research examples presented. For example, the research using cell-based therapies for the treatment of kidney disease highlights the great many challenges encountered and the patience and resources one requires when looking at successfully translating discoveries made in the laboratory into a clinical or human application.

It is clear that research translation is often a very difficult and lengthy process that requires ample resources and planning. The good news is that funding bodies are recognizing this and are committed to dedicating resources to the goal of ensuring that scientific discoveries eventually benefit patients in a timely fashion. Specific to the area of nephrology, the KRESCENT program has a mandate to direct its funds towards a balance of clinical and basic science research, providing a comprehensive training program with an emphasis on translational research and collaboration among investigators enrolled in the program. With enhanced funding and awareness over recent years, the success of research translation should improve over the coming years, ultimately improving the care of patients with kidney disease.

## Abbreviations

AKI: Acute kidney injury
CIHR: Canadian Institutes of Health Research
CKD: Chronic kidney disease
CTSA: Clinical and Translational Science Award
eGFR: Estimated glomerular filtration rate
EOC: Early outgrowth cell
IFT: Intraflagellar transport
IFTA: Intraflagellar transport subcomplex A
JBTS: Joubert syndrome
KFRE: Kidney failure risk equation
KRESCENT: Kidney Research Scientist Core Education and National Training
NCATS: National Center for Advancing Translational Sciences
NGS: Next-Genereation Sequencing
NIH: National Institutes of Health
NPHP: Nephronophthisis
PKD: Polycystic kidney disease
TMEM: Transmembrane
WDR: WD Repeat
